# Serum Monounsaturated Triacylglycerol Predicts Steatohepatitis in Patients with Non-alcoholic Fatty Liver Disease and Chronic Hepatitis B

**DOI:** 10.1038/s41598-017-11278-x

**Published:** 2017-09-05

**Authors:** Rui-xu Yang, Chun-xiu Hu, Wan-lu Sun, Qin Pan, Feng Shen, Zhen Yang, Qing Su, Guo-wang Xu, Jian-gao Fan

**Affiliations:** 1 Center for Fatty Liver, Department of Gastroenterology, Xinhua Hospital, Shanghai Jiao Tong University School of Medicine, Shanghai, 200092 China; 2 CAS Key Laboratory of Separation Science for Analytical Chemistry, Dalian Institute of Chemical Physics, Chinese Academy of Sciences, Dalian, China; 3 Department of Endocrinology, Xinhua Hospital, Shanghai Jiao Tong University School of Medicine, Shanghai, 200092 China

## Abstract

Chronic liver disease is associated with lipid metabolic disruption. We carried out a study to determine serum lipidomic features of patients with non-alcoholic fatty liver disease (NAFLD) and active chronic hepatitis B (CHB) and explored the biomarkers for non-alcoholic steatohepatitis (NASH). Serum lipidomic profiles of healthy controls (*n* = 23) and of biopsy–proven NAFLD (*n* = 42), CHB with NAFLD (*n* = 22) and without NAFLD (*n* = 17) were analyzed by ultra-performance liquid chromatography–tandem mass spectrometry. There were distinct serum lipidome between groups of NAFLD and CHB without NAFLD. Most of the neutral lipids and ceramide were elevated in the NAFLD group but were decreased in the CHB without NAFLD group. Plasmalogens were decreased in both groups. Triacylglycerols (TAGs) with lower carbon numbers and double bonds were increased in subjects with NASH. Serum monounsaturated TAG was a significant predictor of NASH (OR = 3.215; 95%CI 1.663–6.331) and positively correlated with histological activity (r = 0.501; *P* < 0.001). It showed good predictability for NASH in the NAFLD group [area under the receiver operating characteristic curves (AUROC) = 0.831] and was validated in the CHB group (AUROC = 0.833); this characteristic was superior to that of cytokeratin-18 and alanine transaminase. The increase in monounsaturated TAG might be a specific marker for NASH in both NAFLD and CHB patients.

## Introduction

Non-alcoholic fatty liver disease (NAFLD) and active chronic hepatitis B (CHB) are the major burdens of chronic liver diseases in China^1^. Non-alcoholic steatohepatitis (NASH) is the severe form of NAFLD and closely related to liver cirrhosis and hepatocellular carcinoma. The gold standard of diagnosis of NASH depends on liver biopsy. Recent study has shown that serum lipid and molecular metabolites could be used to differentiate between non-alcoholic fatty liver (NAFL) and NASH^2, 3^. The noninvasive strategies for the diagnosis of NASH by lipidomic approach remained great clinical importance.

There is a growing concern regarding the impact of chronic HBV infection including CHB on lipid metabolism and fatty liver^4^. Studies have reported that HBV carrier is associated with a lower prevalence of NAFLD, hypertriglyceridemia, and the metabolic syndrome^5–8^. HBV infection might cause disruption of different aspects of lipid metabolism. The comprehensive evaluation of lipidomic profiles of CHB patients may provide the approach to understand the impact of chronic HBV infection on lipid metabolism.

Recently, concomitant NAFLD is becoming common in patients with CHB and is reported to be the common reason for serum elevation of alanine aminotransferase (ALT) in these patients with low viral load^9^. Hepatocyte injury caused by steatohepatitis and/or virus related to necroinflammation remains difficult to distinguish in patient concurrence with NAFLD and CHB. Studies have reported that the co-existed NASH is related to the severity of liver fibrosis in CHB patients^10^; the detection of NASH is thus essential for such patients. Although various serum markers for the non-invasive diagnosis of NASH have been explored, NASH biomarkers specifically in patients with CHB are still required. In our study, we established an ultra-performance liquid chromatography–tandem mass spectrometry (UPLC-MS/MS) platform to analyze the similarities and differences of serum lipidomic changes in healthy controls, patients with liver biopsy–proven NAFLD, active CHB with and without NAFLD and to explore the NASH-specific serum lipid biomarkers in these patients.

## Materials and Methods

### Study subjects

This study enrolled consecutive healthy adults with normal medical check-up results (control group), liver biopsy–proven patients with NAFLD (NAFLD group), and active CHB patients with and without fatty liver (CHB with NAFLD and CHB without NAFLD groups) between May 2012 and May 2014 in Shanghai Xinhua Hospital. All participants gave a systemic medical history and underwent serum biochemical tests, abdominal ultrasound (Siemens S2000, Siemens Medical Solutions, Erlangen, Germany), and FibroScan-502 (Echosens, Paris, France) evaluations in less than 1 week before liver biopsy. The study was conducted in accordance with the principles of the Declaration of Helsinki and approved by the institutional review board of Xinhua Hospital, and written informed consent was obtained from each enrolled subject.

The inclusion criteria for each group were as follows. Control group: routine health check-up adults with normal results of blood biochemical tests and without HBV-infected markers, normal results of abdominal ultrasound, normal values of controlled attenuation parameter (CAP) (<238 dB/m) and liver stiffness measurement (LSM) (<7.4 kPa) by FibroScan-502. NAFLD group: histological evidence of >5% hepatocytes with macrovesicular or mixed steatosis. CHB group: positive serum hepatitis B surface antigen (HBsAg) and detectable HBV DNA for at least 6 months, with biopsy-proven active chronic viral hepatitis, further divided into subgroups according to with and without hepatic steatosis or NASH.

Exclusion criteria included subjects with excessive alcohol consumption (more than 140 g per week for men and more than 70 g for women) in the past 12 months, age younger than 18 years old, other types of viral hepatitis, primary biliary cholangeitis, autoimmune hepatitis, Wilson’s disease, drug-induced liver injury, liver cirrhosis (F4), hepatocellular carcinoma, diabetes, coronary artery disease, and lack of written consent.

### Clinical and biochemical assessments

Clinical assessments such as age, gender, height, body weight, and body mass index (BMI) were characterized for the study population. Blood sample was collected from each subject after 12-h fasting. Biochemical tests were performed to measure ALT, aspartate aminotransferase (AST), alkaline phosphatase (ALP), and gamma-glutamyl transferase (γ-GT) by standard enzyme methodology, using a multichannel automatic analyzer (Bayer ADVIA 1650, Moss, Norway). Fasting plasma glucose (FPG), total cholesterol (TC), and triacylglycerol (TAG) were analyzed using Wako Bioproducts (Wako Pure Chemical Industries, Richmond, VA, United States). Serum cytokeratin (CK)-18 were measured with a human CK18-M30 enzyme-linked immunosorbent assay (ELISA) kit (U-1197; Peviva, Nacka, Sweden) and a CK18-M65 ELISA Kit (U-2098; Peviva, Nacka, Sweden).

### Working definition of CHB and NASH

Percutaneous liver biopsies were performed and scored by an experienced hepatopathologist, who was blinded to the clinical data. The SAF (steatosis [0–3]; activity, hepatocyte ballooning [0–2] and lobular inflammation [0–2]; fibrosis [0–4]) scoring system was used for the evaluation of NAFLD^11^. The working definition of NASH in NAFLD subjects was based on the joint presence of hepatic steatosis (>5% hepatocytes), hepatocyte ballooning, and lobular inflammation^11^. The histological assessment of chronic HBV infected patients was according to the METAVIR scoring system: histological activity (piecemeal necrosis and lobular necrosis: 0–3) and fibrosis scoring (0–4)^12^. CHB was defined by the typical periportal/portal hepatitis with piecemeal necrosis of hepatocytes, when co-existed >5% hepatocytes steatosis defined as CHB with NAFLD, and then concurrence of hepatic steatosis, hepatocyte ballooning, and lobular inflammation defined as CHB with NASH.

### Sample collection and UPLC-MS/MS analysis

Serum samples were collected after overnight fasting for 12 h, and lipids were extracted following a methyl tert-butyl ether (MTBE)–based extraction^13^. Quality control (QC) samples were obtained by mixing all serum samples and processed following the procedures. UPLC (Waters, Milford, USA) coupled with a Triple TOF 5600 mass spectrometer (AB SCIEX, USA) system was applied to serum lipidomic analysis^14^. Separation of lipids was performed on a UPLC ACQUITY C_8_ BEN column (2.1 mm × 100 mm × 1.7 μm, Waters, Milford, USA). Mobile phases consisted of (A) 60% acetonitrile in water, 10 mM ammonium acetate. (B) 90% isopropanol in acetonitrile, 10 mM ammonium acetate. The flow rate was 0.26 ml/min, with the elution gradient as follows: 32% B was maintained in the first 1.5 min, then increased to 85% B in 14 min, with another linear increase to 97% B from 15.5 to 15.6 min; this was maintained for the next 2.4 min and followed by 32% B in the next 2 min for column equilibration.

Data were obtained in both positive and negative electrospray ionization (ESI) modes. The detailed parameters of mass spectrometry were as follows: ion spray voltage, 4500 V (−) and 5500 V (+); declustering potential: 100 V (−) and 100 V (+); collision energy: 10 V (−) and 10 V (+); curtain gas: 35 psi; interface heater temperature: 600 °C (−) and 500 °C (+).

The data were acquired by Analyst TF 1.6 software (AB SCIEX, Framingham, MA) and were first submitted to LipidView (AB SCIEX, Framingham, MA) for lipid identification. The data were then imported in PeakView (AB SCIEX, Framingham, MA) to create lipid name, M/Z, and retention time. MultiQuant 2.0 (AB SCIEX, Framingham, MA) was used for quantitative analysis. Peak areas were obtained from MultiQuant output. After normalization with corresponding internal standards, the data of all the detected lipids in QCs were evaluated by their relative standard deviation (RSD), and only those with RSD below 30% were retained for further analysis.

### Statistical analysis

The statistical analysis was performed with SPSS software (version 22.0, Chicago, USA). The continuous normally distributed variables were expressed as mean ± standard deviation, and the categorical or non-normal distributed variables were expressed as medians with interquartile range. Student’s *t*-test (for continuous and normally distributed variables), Mann-Whitney U test (non-normal distributed variables), and Chi-square (χ^2^) test (categorical variables) were performed to investigate the differences. Multivariate statistic for lipidomic data was performed with SIMCA-p software (version 13.0, Umetrics AB, Sweden). Orthogonal partial least squares–discriminant analysis (OPLS-DA) was established to analyze serum lipid profile, and a seven-fold cross-validation test was performed for significance testing for OPLS-DA. Receiver operating characteristic (ROC) curves were plotted, and area under the ROC (AUROC) was calculated to compare the accuracy of test; the sensitivity, specificity, positive predictive value (PPV), negative predictive value (NPV), positive likelihood ratios (LR+), and negative likelihood ratio (LR−) for cutoffs were also calculated. Correlations between lipids and histological features were assessed by Spearman’s correlation. *P* values < 0.05 were considered to be statistically significant.

## Results

### General characteristics of the study population

A total of 104 subjects met the inclusion criteria were enrolled in this study, including control group (*n* = 23), NAFLD group (*n* = 42), CHB without NAFLD group (*n* = 17), and CHB with NAFLD group (*n* = 22). The detailed characteristics of these subjects are summarized in Table 1. There was no significant difference in the gender, age, and FPG among the four groups. Compared with the control group, BMI and serum levels of ALT, AST, ALP, γ-GT, and TAG in the NAFLD group were significantly increased. The CHB without NAFLD group had lower BMI and TC and higher levels of ALT, AST, and γ-GT than the control group, whereas BMI, ALT, and AST were significantly increased in the CHB with NAFLD group. Forty-four cases were found to have NASH among NAFLD patients with and without CHB. There was no significant difference in the percentage of NASH subjects between the NAFLD group and the CHB with NAFLD group.

**Table 1:** Characteristics of the study population.

Parameters	Control (*n* = 23)	NAFLD (*n* = 42)	CHB without NAFLD (*n* = 17)	CHB with NAFLD (*n* = 22)	*P* ^a^	*P* ^b^	*P* ^c^
Age (years)	38 (36–43)	34.5 (29–51)	37 (27.5–49.5)	36 (24.75–46.75)	NS	NS	NS
Male (%)	14 (60.8%)	26 (61.9%)	9 (52.94%)	16 (72.7%)	NS	NS	NS
Waistline (cm)	80.96 ± 6.31	91.41 ± 6.96	75.14 ± 8.64	88.83 ± 8.53	<0.001	NS	0.002
BMI (kg/m^2^)	23.80 ± 2.48	27.44 ± 3.20	21.83 ± 2.96	25.52 ± 2.64	<0.001	0.028	0.030
ALP (U/L)	66.0 (55.0–84.0)	84 (62.6–100.6)	76.0 (62.5–96.0)	77.0 (63.3–95.6)	0.020	NS	NS
γ-GT (U/L)	16.2 (11.7–27.6)	60.0 (37.9–89.0)	44.0 (13.5–183.8)	30.5 (21.3–58.3)	<0.001	0.035	0.003
ALT (U/L)	21.0 (17.1–28.4)	64.2 (40.8–111.7)	96.0 (32.5–170.0)	52.6 (23.7–68.8)	<0.001	<0.001	<0.001
AST (U/L)	21.0 (17.0–24.0)	38.5 (25.0–68.5)	58.0 (23.0–92.5)	28.6 (20.4–45.4)	<0.001	<0.001	0.005
TC (mmol/L)	4.44 (4.25–4.97)	4.63 (4.24–5.14)	3.64 (3.19–4.02)	5.11 (3.92–5.65)	NS	<0.001	NS
TAG (mmol/L)	1.25 (0.86–1.64)	1.63 (1.12–2.51)	1.22 (0.78–1.63)	1.26 (1.03–2.11)	0.042	NS	NS
FPG (mmol/L)	5.20 (4.50–5.40)	5.25 (4.52–6.47)	4.85 (4.25–5.37)	5.36 (4.81–5.91)	NS	NS	NS
NASH percentage	—	30 (71.43%)	0 (0%)	14 (63.63%)	—	—	—
Portal inflammation	—	0 (0%)	17 (100%)	22 (100%)	—	—	—

NAFLD, non-alcoholic fatty liver disease; CHB, active chronic hepatitis B; NASH, non-alcoholic steatohepatitis; BMI, body mass index; ALP, alkaline phosphatase; γ-GT, gamma-glutamyl transpeptidase; AST, aspartate aminotransferase; ALT, alanine aminotransferase; TAG: triacylglycerol; TC: total cholesterol; FPG: fasting plasma glucose.

Data are expressed as mean ± Std. Deviation, median (25th-75th percentile), or n (%), as appropriate. *P* ^a^, *P* ^b^, and *P* ^c^ indicate the *P* value of the NAFLD group, CHB without NAFLD group, and the CHB with NAFLD group in comparison with the control group, respectively. The *P* value reflects the statistical significance calculated by Student’s t-test (normally distributed data), Chi-square test (gender), and Mann–Whitney U-test (not normally distributed data). NS: non-significant.

### Changes in serum lipidomic profiles of the study population

A total of 239 lipids were identified in the serum, including neutral lipids (CE: cholesterol ester; DAG: diacylglycerol; TAG: triacylglycerol); phospholipids (PC: phosphatidylcholine; LPC: lysophosphatidylcholine; PE: phosphatidylethanolamine; LPE: lysophosphatidylethanolamine; PI: phosphatidylinositol; LPI: lysophosphatidylinositol); plasmalogens (PC-O: choline plasmalogen and PE-O: ethanolamine plasmalogen; LPC-O: lysophosphatidylcholine plasmalogen; LPE-O: lysophosphatidylethanolamine plasmalogen); sphingolipids (SM: sphingomyelin; Cer: ceramide; HexCer: hexosylceramide), and free fatty acids (Supplementary Table).

The relative standard deviation (RSD) of every lipid in the QC samples was calculated, and 99% of the RSD was <30% (Supplementary Figure); this indicated the stability of the UPLC-MS/MS detection.

**Figure 1:**
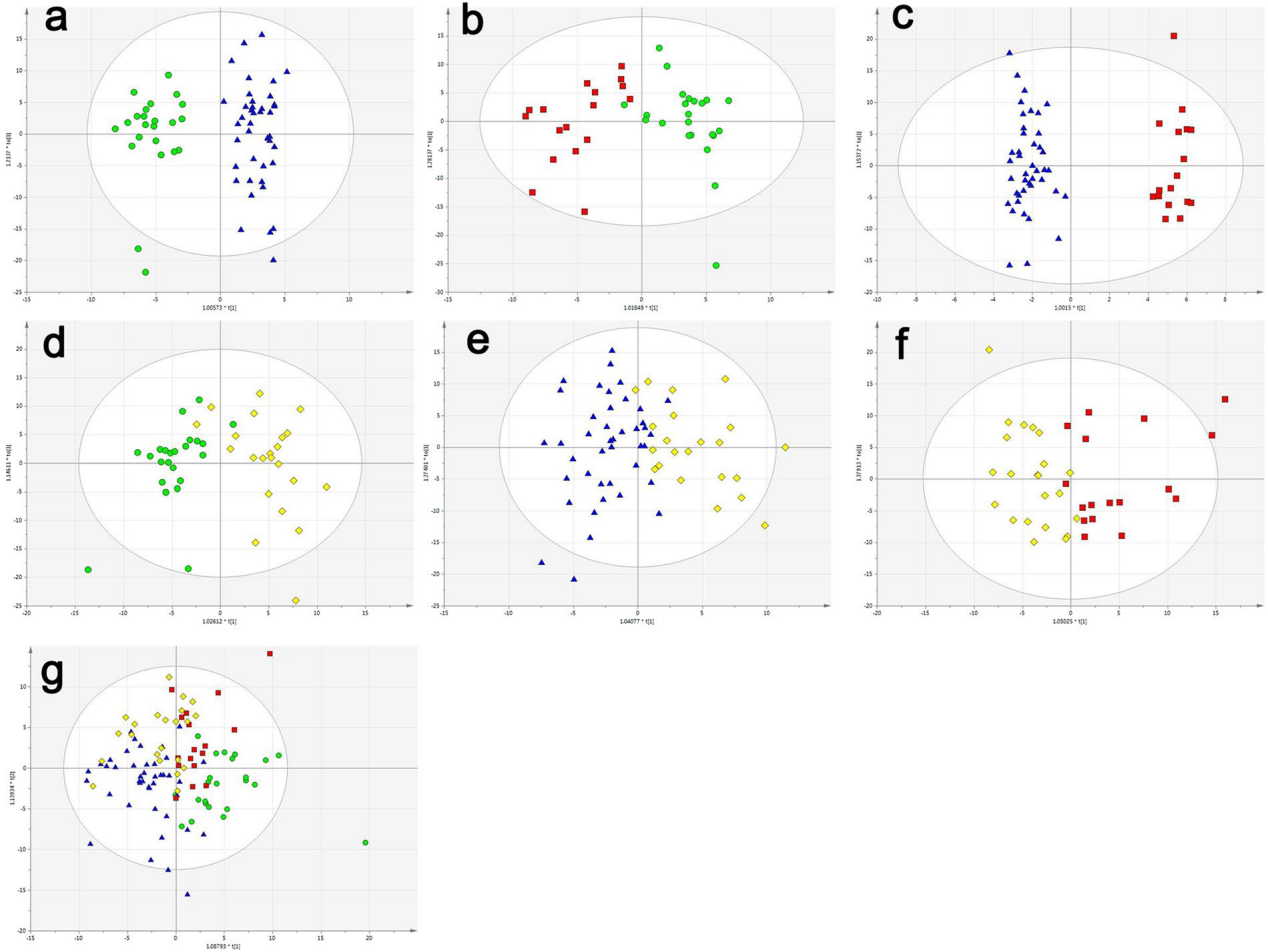
OPLS-DA scores plot of the control group (green circles), the NAFLD group (blue triangles), the CHB without NAFLD group (red squares), and the CHB with NAFLD group (yellow diamonds). (**a**) OPLS-DA scores plot of the control and NAFLD groups: (R2Y = 0.907, Q2 = 0.647) (**b**) OPLS-DA scores plot of the control and CHB without NAFLD groups (R2Y = 0.775, Q2 = 0.518). (**c**) OPLS-DA scores plot of the NAFLD and CHB without NAFLD groups (R2Y = 0.961, Q2 = 0.587). (**d**) OPLS-DA scores plot of the control and CHB with NAFLD groups: (R2Y = 0.729, Q2 = 0.607) (**e**) OPLS-DA scores plot of the NAFLD and CHB with NAFLD groups (R2Y = 0.595, Q2 = 0.254). (**f**) OPLS-DA scores plot of the CHB without NAFLD groups and CHB with NAFLD groups (R2Y = 0.572, Q2 = 0.264). (**g**) Bidirectional orthogonal partial least squares-discrimination analysis (O2PLS-DA) of the four groups. The *P*(CV-ANOVA) were <0.05 for all the OPLS-DA models.

To evaluate the ability to discriminate different groups, OPLS-DA was performed for discrimination between the control group and the NAFLD group; the control group and the CHB without NAFLD group; and the NAFLD group and the CHB without NAFLD group (Fig. 1a–c). The results indicated good predictive performance and repeatability of the group discrimination. However, changes in serum lipidomic profiles in the CHB with NAFLD group were overlapped between those in the NAFLD group and CHB without NAFLD group in the O2PLS-DA analysis (Fig. 1g), and the R2X, R2Y and Q2 value showed indistinctive predictive performance and repeatability of the group discrimination (Fig. 1d–f).

### Distinct lipid alterations between groups of NAFLD and CHB without NAFLD

**Figure 2:**
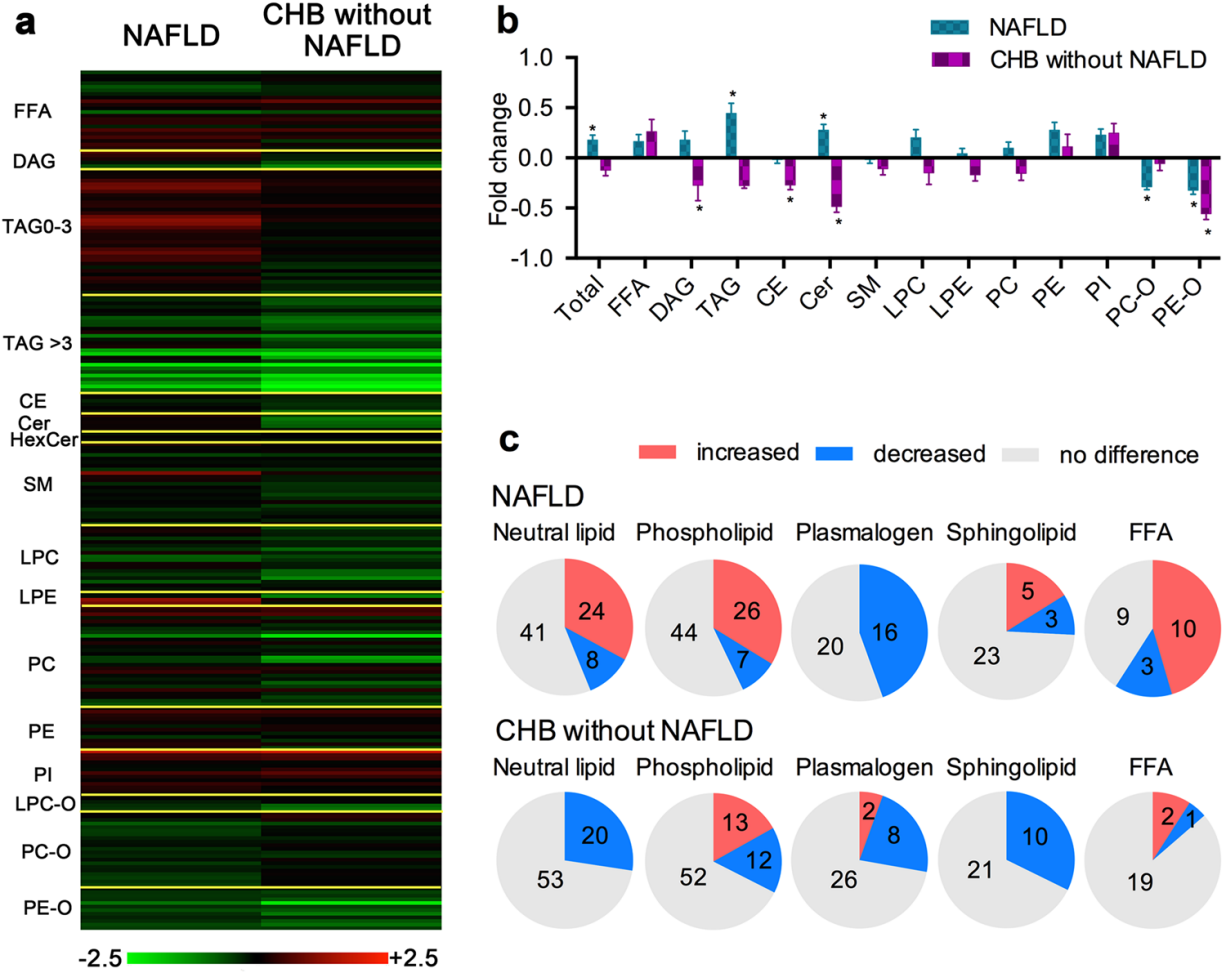
Changes in lipid contents of the NAFLD and CHB without NAFLD groups. (**a**) Complete list of fold changes (log2) for each lipid species in the NAFLD and CHB without NAFLD groups. (**b**) Changes in total serum lipid contents of the NAFLD and CHB without NAFLD, *P < 0.05. (**c**) Significant changes in lipid species within neutral lipids, phospholipids, plasmalogens, sphingolipids, and free fatty acids in serum of the NAFLD (upper panel) and CHB without NAFLD (lower panel) groups.

Compared with the control group, the changes (log2) for 239 species detected in the NAFLD and the CHB without NAFLD groups are shown in Fig. 2a; the findings indicate different alteration trends for each species characterized by their lipid classes, carbon numbers, and double bonds. The change in total lipid content is shown in Fig. 2b. Compared with the control group, the NAFLD group showed markedly increased total lipid and TAG content (*P* < 0.05), which were decreased in the CHB without NAFLD group, but did not reach statistical difference. Similarly, serum DAG and Cer were increased in the NAFLD group and decreased in the CHB without NAFLD group (*P* < 0.05). However, the serum levels of plasmalogens such as PE-O were markedly decreased in both groups (*P* < 0.05), whereas PC-O was mainly decreased in the NAFLD group (*P* < 0.05).

The significantly changed lipid molecules compared with the control group are summarized in Fig. 2c and Table 2. Most of the neutral lipids composed of TAGs, DAGs, and CEs were increased in the NAFLD group but decreased in the CHB without NAFLD group. In agreement with the decreased total plasmalogens, most of the plasmalogens were markedly decreased in both diseases, especially in the NAFLD group. There were bi-directional changes in sphingolipids species in the NAFLD group, whereas all the significantly changed sphingolipids were decreased in the CHB without NAFLD group. Furthermore, compared with the control group, the NAFLD group showed significant alterations in FFAs.

**Table 2:** Significant changed lipid species in NAFLD and CHB without NAFLD.

Lipids	NAFLD/control	*P*	Lipids	NAFLD/control	*P*	Lipids	NAFLD/control	*P*	Lipids	CHB without NAFLD/control	*P*	Lipids	CHB without NAFLD/control	*P*
FFA 12:0	0.765	0.008	TAG 54:0	1.2	0.023	PE 40:5	1.37	0.005	FFA 16:1	2.391	0.01	PC 36:5	0.569	0.018
FFA 16:1	1.781	0	TAG 54:1	2.045	0.01	PI 32:1	3.917	0	FFA 20:0	0.841	0.045	PC 36:6	0.548	0.01
FFA 18:0	0.801	0.002	TAG 54:2	1.694	0.008	PI 34:1	1.635	0	FFA 20:2	1.596	0.026	PC 38:2	1.391	0.038
FFA 18:1	1.262	0.015	TAG 54:7	0.515	0.013	PI 34:2	1.541	0.001	CE 20:4	0.794	0.022	PC 38:6	0.67	0.018
FFA 18:2	1.233	0.032	TAG 54:8	0.466	0.005	PI 36:4	1.625	0	CE 22:6	0.648	0.032	PC 40:4	1.343	0.014
FFA 20:0	0.656	0	TAG 56:8	0.637	0.009	PI 38:3	1.302	0.035	DAG 36:4	0.553	0.004	PC 40:8	0.664	0.004
FFA 20:2	1.456	0.004	TAG 56:9	0.489	0.021	PI 40:4	1.553	0.001	TAG 52:4	0.672	0.045	PE 34:1	1.941	0.048
FFA 20:3	2.08	0.001	TAG 58:5	1.293	0.02	PI 40:5	1.366	0.021	TAG 52:5	0.566	0.026	PI 32:1	4.63	0.001
FFA 20:4	1.799	0.013	TAG 58:10	0.565	0.039	PI 40:6	1.289	0.015	TAG 52:6	0.484	0.021	PI 34:1	1.76	0.007
FFA 20:5	2.7	0.045	TAG 58:11	0.462	0.019	PC-O 34:2	0.634	0	TAG 52:7	0.448	0.012	PI 34:2	1.64	0.007
FFA 22:4	1.725	0	LPC 16:0	1.273	0.015	PC-O 34:3	0.786	0.006	TAG 54:5	0.678	0.021	PI 36:3	1.361	0.011
FFA 22:5	1.719	0.001	LPC 18:2	0.798	0.008	PC-O 36:2	0.73	0.002	TAG 54:7	0.354	0.003	PI 36:4	1.84	0.001
FFA 22:6	1.806	0.011	LPC 18:3	1.274	0.021	PC-O 36:3	0.754	0.008	TAG 54:8	0.331	0.011	PI 38:3	1.456	0.023
DAG 34:1	1.879	0	LPC 20:0	0.702	0.004	PC-O 36:4	0.855	0.036	TAG 56:5	0.759	0.043	PI 40:4	1.339	0.028
DAG 34:2	1.529	0.023	LPC 20:1	0.735	0.035	PC-O 38:5	0.786	0.001	TAG 56:7	0.537	0.025	PC-O 32:0	1.318	0.014
DAG 36:2	1.281	0.04	LPC 20:2	1.345	0.045	PC-O 38:7	0.835	0.02	TAG 56:8	0.541	0.018	PC-O 34:0	1.349	0.015
TAG 40:1	1.476	0.033	LPC 20:5	0.783	0.005	PC-O 40:5	0.748	0.001	TAG 56:9	0.332	0.003	PC-O 34:2	0.709	0.012
TAG 42:1	1.39	0.003	LPC 24:0	0.728	0.008	PC-O 42:5	0.77	0	TAG 58:10	0.33	0.006	PE-O 36:5	0.665	0.005
TAG 42:2	1.512	0.035	LPI 18:0	4.113	0.002	PC-O 42:6	0.752	0.003	TAG 58:11	0.242	0.001	PE-O 36:6	0.359	0
TAG 44:2	1.384	0.003	LPI 20:4	1.976	0	PC-O 44:5	0.786	0.001	TAG 58:4	0.713	0.027	PE-O 38:5	0.751	0.04
TAG 46:0	1.687	0.001	PC 30:0	1.792	0.001	PC-O 44:6	0.74	0	TAG 58:8	0.598	0.003	PE-O 38:6	0.664	0.013
TAG 46:1	1.599	0.035	PC 32:0	1.308	0.024	PE-O 38:6	0.709	0.011	TAG 58:9	0.446	0.005	PE-O 38:7	0.544	0
TAG 48:0	2.307	0	PC 32:1	2.096	0	PE-O 38:7	0.813	0.028	TAG 60:12	0.474	0.012	PE-O 40:7	0.677	0.012
TAG 48:1	2.464	0.001	PC 34:1	1.404	0.001	PE-O 40:8	0.715	0.001	LPC 14:0	0.693	0.015	PE-O 40:8	0.615	0.004
TAG 48:2	1.914	0.004	PC 36:1	1.303	0.005	PE-O 42:7	0.767	0.014	LPC 18:2	0.651	0.001	Cer 40:1:2	0.741	0.015
TAG 50:0	2.575	0.001	PC 38:2	1.3	0.002	Cer 40:1;2	1.362	0.003	LPC 20:4	0.625	0	Cer 40:2:2	0.591	0.001
TAG 50:1	2.673	0	PC 38:3	1.534	0	Cer 42:2;2	1.226	0.047	LPC 20:5	0.659	0.001	Cer 41:1:2	0.656	0.003
TAG 50:2	2.072	0	PC 40:4	1.539	0	SM 32:1;2	1.311	0.001	LPC 22:6	0.529	0	Cer 42:1:2	0.634	0
TAG 50:3	1.55	0.012	PC 40:7	0.747	0.001	SM 34:0;3	0.756	0.004	LPE 22:6	0.633	0.009	SM 36:3:2	0.781	0.016
TAG 52:0	1.823	0.001	PC 40:8	0.675	0	SM 36:0;2	2.429	0	PC 30:0	2.046	0.008	SM 38:1:2	0.802	0.012
TAG 52:1	2.675	0	PE 34:1	1.809	0.001	SM 36:1;2	1.233	0.01	PC 32:0	1.535	0.013	SM 38:2:2	0.815	0.031
TAG 52:2	1.617	0.003	PE 34:2	1.426	0.007	SM 36:3;2	0.819	0.006	PC 32:1	2.079	0.015	SM 40:1:2	0.72	0
TAG 52:3	1.341	0.028	PE 36:1	1.283	0.018	SM 42:1;3	0.783	0.006	PC 34:4	0.724	0.041	SM 40:2:2	0.818	0.011
TAG 52:7	0.638	0.034	PE 36:4	1.35	0.015				PC 34:5	0.492	0.044	SM 42:1:2	0.758	0.003

The lipid species are expressed as name carbon numbers: double bond numbers. The *P* value reflects the statistical significance calculated by Student’s t-test.

### TAGs with lower carbon numbers and double bonds in NASH patients

**Figure 3:**
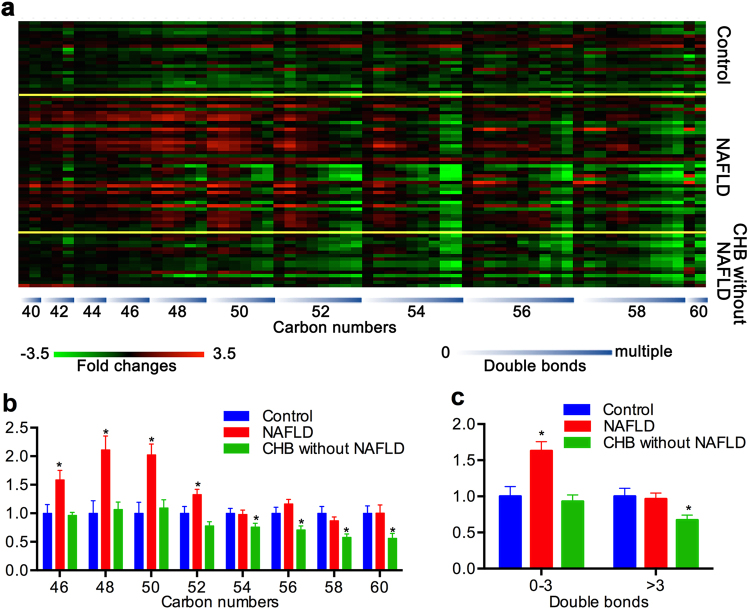
Change in TAG molecules with specific carbon numbers and double bonds in the NAFLD group and CHB without NAFLD group. (**a**) Fold changes of each TAG molecule in each patient (carbon numbers from 40 to 60, double bonds from 0 to multiple). (**b**) The TAGs with lower carbon numbers increased in the NAFLD group, and the TAGs with higher carbon numbers decreased in the CHB without NAFLD group. (**c**) The TAGs with 0–3 double bonds increased in the NAFLD group, whereas the TAGs with more than 3 double bonds increased in the CHB without NAFLD group.

The changes of TAG species with specific carbon numbers and double bonds in patients with NAFLD and CHB without NAFLD, compared with the control group, are shown in Fig. 3a. The figure shows a ladder pattern wherein from the left to the right, the TAGs decreased with the increase of carbon numbers; within each specific carbon number, however, the TAGs decreased with the respect to the number of double bonds in both the NAFLD group and the CHB without NAFLD group. However, there was an obvious increase in TAGs with lower carbon numbers and double bonds in the NAFLD group. As shown in Fig. 3b,c, the NAFLD group were mainly characterized by the increased TAGs of lower carbon numbers (≤52) and double bonds (0–3) (*P* < 0.05), whereas the CHB without NAFLD group was mainly characterized by decreased TAG with higher carbon numbers (>52) and double bonds (>3) (*P* < 0.05).

**Figure 4:**
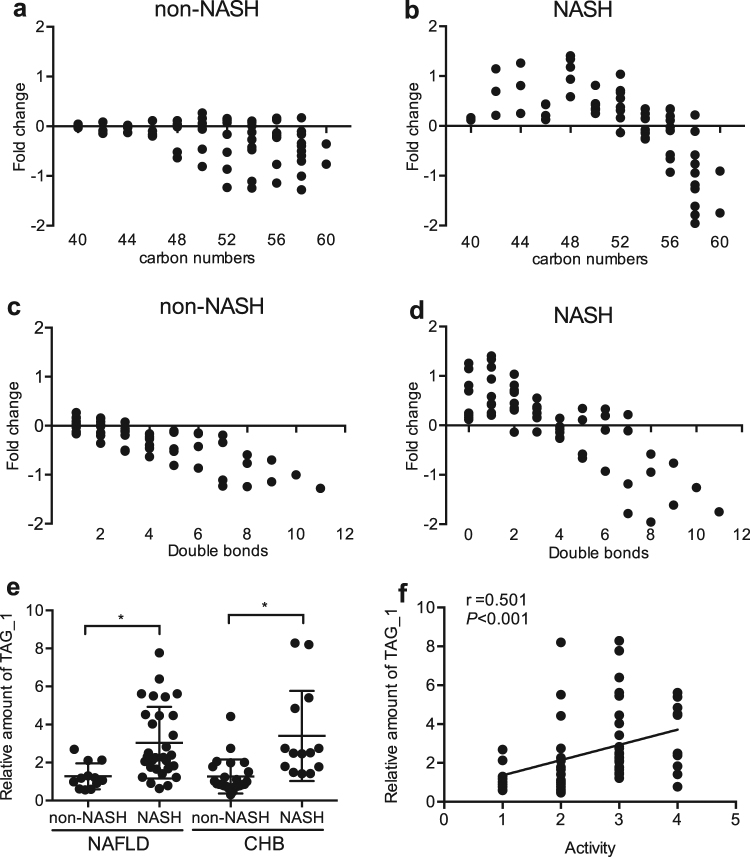
TAG carbon numbers and double bonds in the non-NASH subgroup (*n* = 12) and NASH subgroups (*n* = 30), and the elevation of monounsaturated TAG (TAG_1) is associated with NASH activity. (**a**) The alterations in TAGs with different carbon numbers in the non-NASH subgroup. (**b**) The alterations in TAGs with different carbon numbers in the NASH subgroup. (**c**) The change in TAGs with different double bonds in the non-NASH subgroup. (**d**) The change in TAGs with different double bonds in the NASH subgroup. (**e**) Relative TAG_1 levels in the non-NASH and NASH subgroups in NAFLD and CHB. (**f**) Spearman correlation analysis between TAG_1 levels and hepatic histological activity score.

According to the histological changes, the NAFLD group was divided into a non-NASH subgroup (*n* = 12) and NASH subgroup (*n* = 30). The fold change (compared with the control group) of TAG with specific carbon numbers and double bonds was calculated. As shown in Fig. 4a–d, the NASH subgroup had elevated TAGs with lower carbon numbers and double bonds than the non-NASH subgroup.

### Serum monounsaturated TAG predicts NASH both in NAFLD and CHB patients

We identified the value of TAGs in the diagnosis of NASH in the NAFLD (*n* = 42) and CHB subjects (*n* = 39) by the logistic regression model. After adjusting for BMI, age, gender, and ALT, monounsaturated TAG (TAG_1) was shown to be a significant predictor for NASH (adjusted odds ratio = 3.215; 95% CI: 1.663–6.331; *P* = 0.001). TAG_1 was significantly increased in NASH patients compared with that in non-NASH subjects in both the NAFLD and CHB groups (Fig. 4e), and was positively correlated with a hepatic histological activity score by Spearman correlation analysis in the NAFLD (*n* = 42) and CHB with NAFLD (*n* = 22) subjects (Fig. 4f).

**Figure 5:**
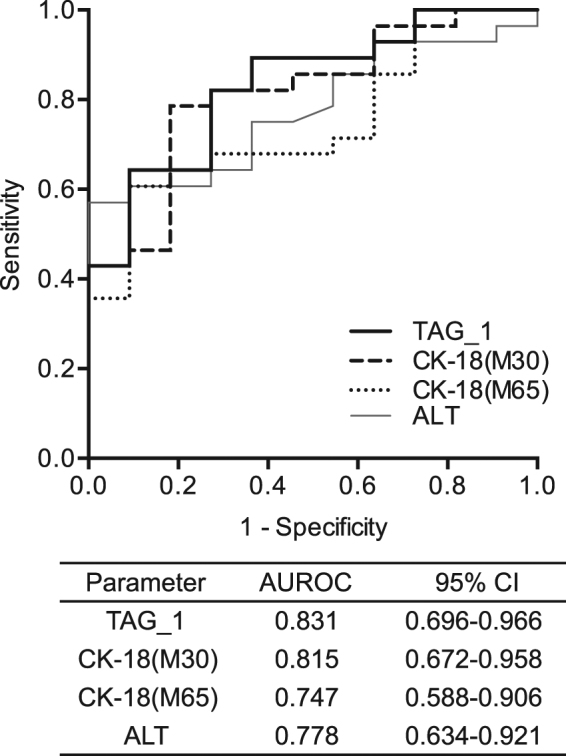
The ROC analyses of TAG_1, CK-18 (M30 and M65), and ALT in the NAFLD group. AUROC: area under the receiver operating characteristic curve; CI, confidence interval.

The ROC analysis for the detection of NASH in the NAFLD group showed that TAG_1 presented good diagnostic value similar to that of CK-18 M30 and better than that of CK-18 M65 and ALT (Fig. 5). The optimal cutoffs were derived according to the Youden index for each parameter. Validations of the performance for the diagnosis of NASH in the CHB group by using established cut-offs are shown in Table 3. TAG_1 showed superior AUROC (0.833 at lower cut-off and 0.774 at higher cut-off) compared with CK-18 M30, CK-18 M65, and ALT in the CHB validation group. TAG_1 also showed good specificity, PPV, NPV, and LR + at higher cut-offs, and good sensitivity, NPV, and LR at lower cut-offs. However, CK-18 M30, CK-18 M65, and ALT showed poor performance in prediction of NASH in CHB patients.

**Table 3:** Validations of the performance for the diagnosis of NASH in CHB patients by using established cutoffs.

Test	Cut-offs	AUROC (95% CI)	Sensitivity	Specificity	PPV	NPV	LR+	LR−
TAG_1	2.1298	0.774 (0.602–0.946)	0.643	0.916	0.818	0.814	7.714	0.390
TAG_1	1.355	0.833 (0.698–0.969)	1	0.708	0.667	1	3.428	0
CK-18 (M30)	251.67	0.655 (0.466–0.843)	0.643	0.681	0.562	0.750	2.020	0.524
CK-18 (M65)	721.52	0.393 (0.203–0.583)	0.214	0.636	0.272	0.56	0.589	1.235
ALT	69.75	0.405 (0.212–0.597)	0.285	0.6	0.285	0.6	0.714	1.190

CHB, active chronic hepatitis B; NASH, non-alcoholic steatohepatitis; TAG: triacylglycerol; CK-18, cytokeratin-18; ALT, alanine aminotransferase; PPV, positive predictive value; NPV, negative predictive value; LR+, positive likelihood ratio; LR−, negative likelihood ratio.

## Discussion

Previous studies revealed lipidomic signatures in the liver and the serum of patients with NAFLD^15–17^, and its possibility of non-invasive approach diagnosing NASH^2, 3^. However, there were few studies of serum lipidomic profiles in CHB and of the biomarkers in the diagnosis of NASH in patients with active chronic viral hepatitis. In our study, we performed the UPLC-MS/MS lipidomic analysis and demonstrated an inverse alteration of serum lipid profile and TAG signatures in the NAFLD and active CHB patients, revealing that the elevation of serum TAGs with one double bond was the feature of NASH with good diagnostic value in NAFLD patients and validated in CHB patients.

One of the notable findings was the opposite lipidomic alterations in patients with NAFLD and CHB without NAFLD in this study. As we expected, there were significant elevations of serum total lipids, neutral lipids, and ceramide in the NAFLD group. NAFLD is featured by the hepatic lipid accumulation of TAG. Generally, insulin resistance plays a key role in the imbalance of hepatic lipids, and TAG accumulation in the liver exacerbates hepatic insulin resistance and increases insulin-antagonizing cytokines in turn^18^. The increased DAG and ceramide could mediate insulin resistance, oxidative stress, apoptosis, and endoplasmic reticulum stress, which are associated with lipotoxic liver injury in NASH^19–22^. Interestingly, our study found decreased serum total lipids, neutral lipids (TAG, DAG, CE), and sphingolipids (Cer, SM) in the CHB without NAFLD group, indicating that chronic active HBV infection may be inversely associated with hyperlipidemia and insulin resistance and may play beneficial roles on lipid profiles^20, 21, 23, 24^. Increasing epidemiological studies have shown that chronic HBV infection is inversely associated with the metabolic syndrome and its some components^6–8, 25, 26^. Chronic HBV infection was associated with lower serum TAG but higher serum levels of adiponectin and visfatin^27, 28^. Our study provided further evidence of the inverse relationship of chronic HBV infection with metabolic factors by lipidomic approach.

Another intriguing finding is that most of the serum plasmalogens were significantly decreased both in the NAFLD and CHB without NAFLD groups. Plasmalogens are a class of membrane phospholipid with a vinyl-ether bond at sn-1 position and protect polyunsaturated fatty acid from oxidative damage, thus acting as sacrificial endogenous oxidants^29, 30^. One study reported that the administration of the plasmalogen precursor alkyl glycerol prevented hepatic steatosis and NASH and an increase in fatty acid oxidation in an animal model^31^. The decreased plasmalogens (PC-O, PE-O) in the NAFLD and CHB without NAFLD groups might be associated with decreased antioxidant capacity in chronic systemic inflammation in our study.

As the major components of neutral lipids, TAG showed distinct patterns in the NAFLD and CHB without NAFLD groups. The decrease in TAGs with higher carbon numbers and double bonds was associated with the decrease in long-chain polyunsaturated fatty acids (LCPUFA) both in NAFLD subjects and CHB without NAFLD subjects. The lower level of LCPUFA is associated with low oxidative status and desaturase enzyme deficiency in chronic liver diseases^32^. However, we demonstrated a stepwise increase in TAGs with lower carbon numbers and double bond only in the NAFLD group, and these increased TAGs were mainly observed in NASH subjects but not in non-NASH subjects. These TAGs were predominantly composed of saturated and monounsaturated fatty acids and they were reported to be associated with an increased risk of insulin resistance and type 2 diabetes^33^.

Finally, the most important finding of the study was that the increase in TAG_1 was the specific lipidomic features of NASH and positively correlated with the hepatic histological activity score. TAG_1 showed good diagnostic value for NASH in the NAFLD patients. In the validation, by using the derived cut-offs in the CHB patients, TAG_1 showed superior predictability for NASH in CHB patients to that of serum CK-18 or ALT, which could hardly diagnose NASH in CHB patients. The concurrence of NASH is the common cause for ALT elevation in CHB patients^9^, and the concomitant NASH could increase the risk of liver fibrosis in CHB patients^10^; the identification of NASH is thus essential for chronic HBV infected patients. Although CAP measured by Fibroscan could evaluate the degree of hepatic steatosis in CHB patients^34–36^, few biomarkers could specifically diagnose NASH in such patients due to the disturbance possibly by HBV related elevation of ALT and the inflammatory status. The increase in TAG_1 reflected the increase in monounsaturated fatty acids and desaturase activity, which are associated with the pathophysiological process of NASH^37^. Our NASH-specific TAG_1 might serve as a specific biomarker for the diagnosis of NASH in NAFLD patients regardless of HBV infection and have potential to distinguish the etiology of abnormal ALT elevation for the appropriate treatment in chronic HBV infected patients with NAFLD.

There were several limitations in our study. We analyzed only the serum lipid profiles rather than simultaneously evaluating the liver tissue. This was based on our consideration that Puri *et al*. has already verified that the circulation lipidomic profile could reflect the liver metabolic status in NASH^15, 16^, and the serum analysis could provide non-invasive biomarkers for the diagnostic application. Second, we did not perform liver biopsy in the control group to verify the diagnosis because of the lack of biopsy indications and ethics principles. To minimize the impact of these limitations, we performed strict medical history taking, biochemical and virological tests, ultrasound, and Fibroscan to rule out the combination of steatosis and other liver diseases. Third, this is a case-control study with a small number of biopsy-proven patients with NAFLD and active chronic HBV hepatitis, inactive HBV carrier (both immunotolerant and after seroconversion) and patients with liver cirrhosis were not included in this study. Fourth, it is interesting to find that there were lower serum AST and ALT levels in the CHB with NAFLD group than that of the CHB without NAFLD group. Though there were no significant difference in the serum HBV DNA load and HBeAg positive rates between the two groups, more severe periportal/portal inflammation in the histological assessment and higher METAVIR activity score were found in CHB without NAFLD group compared with the CHB with NAFLD group, which may explain the higher levels of AST and ALT in the CHB without NAFLD group. Finally, the definition of NASH among patients with CHB is a controversial issue without known criteria. Therefore, it would be difficult to make a solid conclusion on the usefulness of serum monounsaturated TAG for identifying NASH from NAFLD or CHB.

In summary, this study revealed distinct serum lipidomic changes between NAFLD patients and CHB without NAFLD patients, and the notable increase in serum TAG_1 showed good diagnostic performance in patients with NAFLD and even superior accuracy to that of serum CK-18 and ALT in diagnosis of NASH in CHB patients. These results provided evidence of the effect of chronic active HBV infection on lipid metabolism and explored potential non-invasive biomarkers for NASH applicable in CHB patients. Large sample study including inactive HBV carrier and cirrhotic patients will be required to be verified for the NASH-specific biomarkers in the future.

## Electronic supplementary material

Supplementary table and figure
