# Supplementary material for: Serum Monounsaturated Triacylglycerol Predicts Steatohepatitis in Patients with Non-alcoholic Fatty Liver Disease and Chronic Hepatitis B
Source: Sci Rep. 2017 Sep 5;7:10517. doi: 10.1038/s41598-017-11278-x (PMC5585331; doi:10.1038/s41598-017-11278-x)
Supplement: Supplementary file 1 — Supplementary table and figure [file 41598_2017_11278_MOESM1_ESM.pdf]

Serum Monounsaturated Triacylglycerol Predicts Steatohepatitis in Patients with Non-alcoholic Fatty Liver Disease and Chronic Hepatitis B

Short title: Serum Monounsaturated Triacylglycerol Predicts NASH

Rui-xu Yang[1], Chun-xiu Hu[2], Wan-lu Sun[1], Qin Pan[1], Feng Shen[1], Zhen Yang[3], Qing Su[3], Guo-wang Xu[2] & Jian-gao Fan[1]

[1]Center for Fatty Liver, Department of Gastroenterology, Xinhua Hospital, Shanghai Jiao Tong University School of Medicine, Shanghai 200092, China

[2]CAS Key Laboratory of Separation Science for Analytical Chemistry, Dalian Institute of Chemical Physics, Chinese Academy of Sciences, Dalian, China

[3] Department of Endocrinology, Xinhua Hospital, Shanghai Jiao Tong University School of Medicine, Shanghai 200092, China

Corresponding author:

Professor Jian-gao Fan (email: fanjiangao@xinhuamed.com.cn) or Professor Guo-wang Xu (email: xugw@dicp.ac.cn)

Supplementary Table. Lipid classes detected in UPLC-MS/MS profiling

Lipid class	Ion mode	Lipid classes	Number
Neutral lipid	Positive	Cholesterol ester (CE)	6
		Diacylglycerol (DAG)	5
		Triacylglycerol (TAG)	62
Phospholipid	Positive	Phosphatidylcholine (PC)	28
		Lysophosphatidylcholine (LPC)	16
		Phosphatidylethanolamine (PE)	12
		Lysophosphatidylethanolamine (LPE)	4
	Negative	Phosphatidylinositol (PI)	12
		Phosphatidic acid (PA)	1
		Lysophosphatidylinositol (LPI)	2
Plasmalogen	Positive	Choline plasmalogen (PC-O)	21
		Ethanolamine plasmalogen (PE-O)	12
		Lysophosphatidylcholine plasmalogen (LPC-O)	3
		Lysophosphatidylethanolamine plasmalogen	2
Sphingolipid	Positive	Sphingomyelin (SM)	23
		Ceramide (Cer)	5
		Hexosylceramide (HexCer)	3
Free fatty acid	Negative	Free fatty acid (FFA)	22

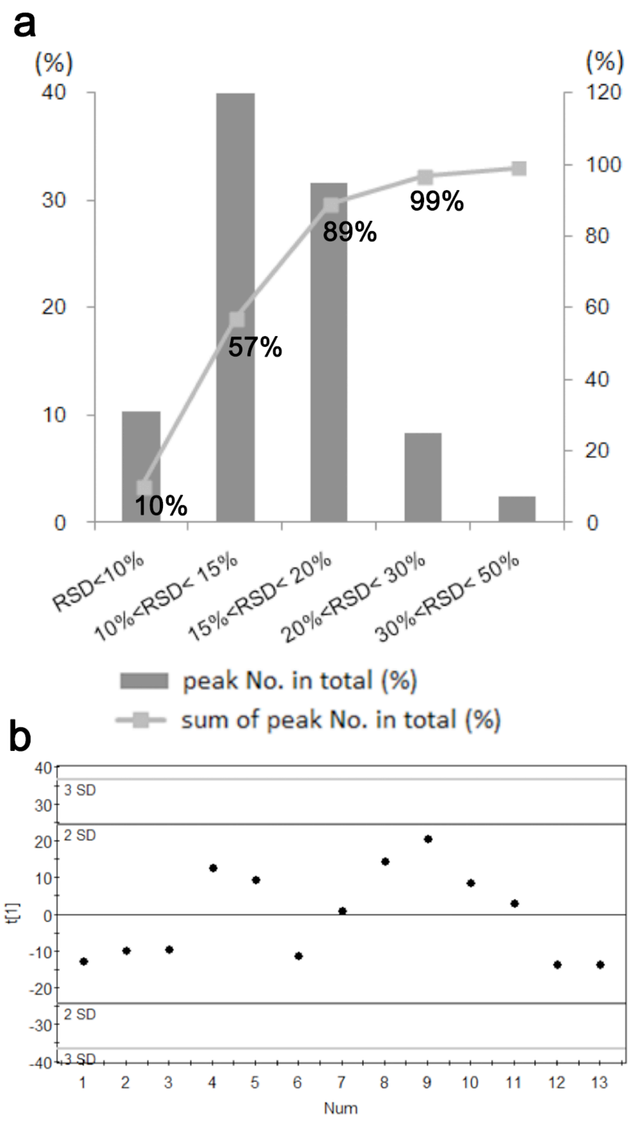

Supplementary Figure. Distribution of RSD in relative content of all peaks in QC samples (**a**) Percentage of peaks of different RSD distribution in QC samples. (**b**) Dynamic distribution of 13 QC samples throughout the analysis.
